# *“We’re just winging it*”. Identifying targets for intervention to improve the provision of hearing support for residents living with dementia in long-term care: an interview study with care staff

**DOI:** 10.1080/09638288.2023.2245746

**Published:** 2023-08-29

**Authors:** Hannah Cross, Christopher J. Armitage, Piers Dawes, Iracema Leroi, Rebecca E. Millman

**Affiliations:** a Manchester Centre for Audiology and Deafness, School of Health Sciences, The University of Manchester, Manchester, UK; b Manchester Centre for Health Psychology, The University of Manchester, Manchester, UK; c Manchester University NHS Foundation Trust, Manchester Academic Health Science Centre, Manchester, UK; d NIHR Greater Manchester Patient Safety Translational Research Centre, The University of Manchester, Manchester, UK; e NIHR Manchester Biomedical Research Centre, Manchester University Hospitals NHS Foundation Trust, Manchester Academic Health Science Centre, Manchester, UK; f Centre for Hearing Research (CHEAR), School of Health and Rehabilitation Sciences, University of Queensland, Saint Lucia, Australia; g Global Brain Health Institute and School of Medicine, Trinity College Dublin, Dublin, Ireland

**Keywords:** Residential care, hearing loss, Behaviour Change Wheel, theoretical domains framework, qualitative research

## Abstract

**Purpose:**

Hearing loss and dementia are common in long-term care home (LTCH) residents, causing communication difficulties and worsened behavioural symptoms. Hearing support provided to residents with dementia requires improvement. This study is the first to use the Behaviour Change Wheel (BCW) to identify barriers and propose interventions to improve the provision of hearing support by LTCH staff.

**Methods:**

Semi-structured interviews with 10 staff members were conducted. Transcripts were analysed according to the BCW’s Theoretical Domains Framework alongside reflective thematic analysis. Relevant intervention functions and exemplar interventions were proposed.

**Results:**

Staff believed hearing support to be beneficial to residents (*Beliefs about Consequences*) but lacked knowledge of hearing loss management (*Knowledge*). Poor collaborations between LTCHs and audiology (*Environmental Context and Resources*), led to despondency, and apprehension about traditional hearing aids for residents (*Optimism*). Despite feeling responsible for hearing support, staff lacked personal accountability (*Social/Professional Role and Identity*).

**Conclusions:**

Future interventions should include staff *Training* (on hearing support), *Education* (on the consequences of unsupported hearing loss), *Enablement* (dementia-friendly hearing devices), *Incentivisation* and *Modelling* (of Hearing Champions) and *Environmental Restructuring* (flexible audiology appointments to take place within the LTCH). Interventions should be multi-faceted to boost the capabilities, opportunities and motivations of LTCH staff.

## IMPLICATIONS FOR REHABILITATION

Hearing support for care home residents with dementia:

- Long-term care staff report inadequate knowledge and awareness of how to support residents’ hearing needs and a lack of personal accountability for providing hearing support.
- They also report poor collaborations with audiologists and apprehension about traditional hearing aids.
- Barriers to hearing support stem from gaps in the capabilities, opportunities and motivations of staff, therefore, interventions should be designed to target all three constructs.
- Interventions to aid hearing support provision should target staffs’ education, training, enablement, persuasion, modelling, incentivisation and environmental restructuring to boost staff capabilities, opportunities and motivations to provide hearing support.

## Introduction

In the United Kingdom, around 70% of long-term care home (LTCHs) residents have dementia [1] and 85% have hearing loss [2]. Concurrent dementia and hearing loss is common [3] and negatively impacts residents’ communication abilities [4], exacerbates agitation and confusion [5], increases loneliness [6] and social withdrawal [7]. It also affects the ability of LTCH staff to provide high-quality care [8]. Addressing the hearing needs of residents with dementia and hearing loss (rereferred to as “residents” in this paper) is therefore essential in improving outcomes.

Hearing support in LTCHs can include hearing aids [9], personal sound amplification devices [7], visual aids [10], staff communication techniques [11] and environmental modifications [12]. Residents can benefit from hearing support as it can reduce their agitation and social isolation and improve quality-of-life [13].

However, providing good-quality hearing support within LTCHs can be challenging [14,15]. For example, residents – particularly those with dementia – may not own or wear hearing devices [9,16] and staff may have difficulties recognising whether residents’ communication difficulties are caused by dementia or hearing loss [8].

Most residents rely on caregivers to meet their hearing needs [17]. Large-scale surveys reveal that LTCH staff lack knowledge, confidence and skills in this area [14,18]. Improving staff knowledge of hearing aids maintenance via training may improve hearing support practices [19]. However, other studies have found that despite staff reporting that they have the confidence and basic skills to manage residents’ hearing loss, rates of hearing device use remain as low as 14% [9,20]. Hearing support provision may therefore be influenced by factors other than staff’s knowledge and skills, for example access to resources or personal motivations.

This semi-structured interview study aims to understand the individual, organisational and systemic barriers and facilitators faced by LTCH staff when providing hearing support to residents. This study is the first to use the Behaviour Change Wheel (BCW) [21], a well-established framework used to develop evidence-led behaviour change interventions, to do so. The BCW includes the Capabilities, Opportunities and Motivations model of Behaviour (COM-B) and the Theoretical Domains Framework (TDF) [22] which are used to understand what needs to change to change people’s behaviour. In the present case, behaviour is the provision, by staff, of hearing support to residents. Thus, this study will look beyond staff Capabilities (e.g., knowledge and skills) and also consider their Opportunities to provide hearing loss support (e.g., time to engage with hearing interventions), and their Motivations (e.g., feelings of professional responsibility). The study will also identify exemplar interventions from the BCW that may be utilised within LTCHs to facilitate staff behaviour change and improve hearing support for residents.

### Research questions

1. What are the barriers and facilitators to the provision of hearing support to LTCH residents with dementia?
2. What are the exemplar interventions with the potential to improve hearing support for LTCH residents with dementia?

## Materials and methods

### Design

Interview schedule development (Appendix A) was guided by COM-B [21]. Questions were designed to capture staff physical capability (physical skills), psychological capability (knowledge), physical opportunity (resources), social opportunity (social cues), reflective motivation (goals) and automatic motivation (emotional drivers) when providing hearing support. Probes derived from the 14-domain TDF [22] explored staff capabilities, opportunities and motivations further [23]. For example, TDF’s “Knowledge” further explored “Psychological Capability”. This framework allows for intervention development informed by an extensive understanding of the barriers and facilitators of the target behaviour.

### Participants

LTCH staff involved in the care of residents were invited to take part. Purposive sampling was used to recruit participants with varied roles, experience and personal demographics.

Participants were recruited as part of a UK-wide online survey study of the capabilities, opportunities and motivations of LTCH staff in providing hearing support for residents [24]. Recruitment was aided by the National Institute for Health and Social Care Research’s ENabling Research In Care Homes (ENRICH) Network, who distributed the study advertisement to “research ready” LTCHs. The advert was also distributed on social media and via word-of-mouth. Survey participants who expressed interest in taking part in the follow-up interview provided their contact email address.

The appropriate sample size, and decision about when to end data collection, was guided by continuous assessment of information power [25]. This included assessment of the richness and quality of participant responses and relevance of responses to the research questions. By the eighth participant, no new information that addressed the research questions was being collected, no new Theoretical Domains were being coded or new themes generated, despite interviews being in-depth, rich in quality and including participants in different job roles. The final two interviews (*n* = 10) ensured that an adequate sample size had been reached.

### Procedure

Ethical approval was obtained from The University of Manchester Research Ethics Committee (2020-10261-16439). All participants provided written informed consent and received remuneration (£15 e-voucher).

Data collection took place between February 2021–May 2021 by one researcher (HC). During interviews, HC introduced herself, provided an overview of the study and reminders of ethical issues, then conducted the interview according to the open-ended schedule. Interviews were conducted on Zoom, recorded using Zoom’s audio-recording function. Recordings were transcribed verbatim into a Microsoft Word document, proofread for accuracy and anonymised by removing mentions of participant, resident, LTCH and company name prior to analysis.

### Analysis

To improve reliability and accuracy, two researchers (HC, REM) were involved in the qualitative analysis of all transcripts. They familiarised themselves with the content of the transcripts before following the coding framework below.

First, deductive coding using direct summative content analysis [26] was used to identify instances of the Theoretical Domains in the transcripts. Identification of a prominent domain was based on the frequency of coding (in ≥60% of transcripts) and emphasis placed on it as a barrier or facilitator by participants, an approach used in qualitative TDF work [27,28]. After coding each transcript independently, the researchers (HC, REM) compared their coding counts of the Domains. Inconsistencies were discussed and resolved to ensure coding was agreed upon. Consistency between coders, assessed by Cohen’s Kappa [29] as recommended by Atkins et al. [23] was substantial (>0.6), therefore a third coder was unnecessary. Prominent Theoretical Domains were mapped onto COM-B domains [23].

An inductive approach was then applied using reflective thematic analysis [30], where themes were generated by one researcher (HC) to further explore the specifics which influenced the provision of hearing support. Themes were assigned to the relevant Theoretical Domains identified in the first-level ­coding stage.

## Results

Participants (*N* = 10) were staff working across eight different LTCHs (Table 1 outlines participant demographics) with a mean of 13.1 years (SD= 7.7) in the care profession. Eight participants took part in one-to-one interviews and two participants (LTCH Manager and Deputy Manager) did their interview together. Interviews lasted approximately 55 min.

**Table 1.** Participant demographics (*N* = 10).

Variables	*N* (%)
Gender	
Female	7(70%)
Male	3(30%)
Ethnicity	
White British	8(80%)
Asian/Asian British	2(20%)
Job role	
Care Assistant	3(30%)
Senior Care Assistant	2(20%)
Registered General Nurse	1(10%)
Registered Mental Health Nurse	1(10%)
Therapy Assistant	1(10%)
Deputy Manager	1(10%)
Home Manager	1(10%)
Years in profession	*Mean* = 13.1 years (*SD* = 7.70)
Fewer than 5 years	2(20%)
5–10 years	1(10%)
10+ years	7(70%)
LTCH registration	
Residential Care Home	4(40%)
Care Home with Nursing	4(40%)
Dementia Specialist Care Home	1(10%)
Don’t Know	1(10%)
Number of Residents in LTCH	*Mean* = 35 (*SD* = 13.65)
Fewer than 21	1(10%)
21–40	5(50%)
40+	4(40%)
LTCH Funding	
Private Company	8(80%)
Local Authority	2(20%)

Care Assistants – provide personal care, Senior Carers – have additional responsibilities such as care planning, Registered Nurses – administer medication and provide clinical care, LTCH Managers – supervise staff, liaise with external health and social care services, Deputy Managers – typically Registered Nurses, who are also involved in managerial duties. Residential Care Home – accommodation, meals, personal care and support provided. Nursing Home (or Care Home with Nursing) – registered nurses for residents with complex health needs also employed. Dementia Specialist Homes – dementia care for residents with advanced cognitive and behavioural needs. Local Authority owned – LTCHs owned by the UK local district, borough or county council.

**Figure 1.**
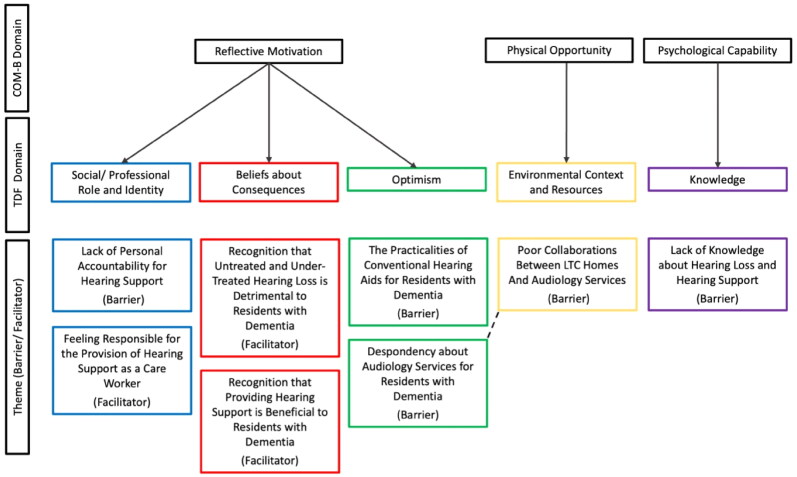
Barriers and facilitators to providing hearing loss support to residents with dementia. Results are organised according to COM-B and TDF domains. Dashed lines represent interacting themes.

Five Theoretical Domains were prominent (Figure 1): Knowledge (identified in 77.8% of interviews), Environmental Context & Resources (88.9%), Social/Professional Role & Identity (77.8%), Optimism (66.7%) and Beliefs about Consequences (100%). Exploratory themes are outlined in the context of each TDF domain. Relevant BCW [21] intervention functions and exemplar interventions are presented in Table 2.

**Table 2.** Summary of key findings, including intervention functions and exemplar interventions, based on the prominent theoretical domains/COM-B domains.

COM Domain	TDF Domain	Barrier/ Facilitator Themes	Exemplar Quotes	Participants coded under this domain	Intervention Functions	Exemplar Interventions
Psych Capability	Knowledge	Lack of Knowledge about Hearing Loss and Hearing Support (Barrier)	I don’t really know what to do with these hearing aids. How am I meant to clean them? What am I meant to clean? What bits can I take apart?… we don’t really ever get told any of these things. – Nurse-1 (23 years in profession)	77.8%	Education Training Education Environmental Restructuring Environmental Restructuring Education Training	Providing easily accessible educational videos and e-learning to staff on how to maintain hearing aids, flashcards and use communication techniques. Placing posters/instruction manuals on how to manage hearing devices, use flashcards and communication techniques in residents’ bedrooms or care plans. Having a “hearing aid box” with all tools to manage hearing aids within the LTCH. Providing hands-on training sessions for LTC staff on how to manage and use hearing devices, use communication techniques and other communication tools.
Reflective Motivation	Social/Professional Role and Identity	Lack of Personal Accountability for Hearing Support (Barrier)Feeling Responsible for the Provision of Hearing Support as a Care Worker (Facilitator)	There’s no one… everyone…tries to kind of…help with it [hearing aid] and help to do it but no one really takes onus for whose job role it is to encourage him [resident] to use it. *–* Therapy Assistant (1.5 years in profession)	77.8%	Incentivisation Modelling	Appointing paid “hearing champions” within the LTCH to take personal ownership for the provision of hearing support to residents with dementia. Having the “hearing champions” as role models for other LTC staff.
	Optimism (pessimism)	The Practicalities of Conventional Hearing Aids for Residents with Dementia (Barrier)Despondency about Audiology Services for Residents with Dementia (Barrier)	Just for residents wearing them [hearing aids], for some of them it possibly is just the stress of having them put on, if they don’t like to be touched, can be more of a hindrance than actually the benefit of actually being able to hear better. – Nurse-1 (23 years in profession)	66.7%	Education Enablement	Educating staff on how to implement a slow, transitionary period for hearing device uptake for residents with dementia. Making adaptations to the hearing devices to be as “dementia-friendly” as possible e.g., using larger hearing devices that require less manual dexterity, using devices that sit over the ears instead of inside the ears.
	Beliefs about Consequences	Recognition that Providing Hearing Support is Beneficial to Residents with Dementia (Facilitator)Recognition that Untreated and Under-Treated Hearing Loss is Detrimental to Residents with Dementia (Facilitator)	She’ll be able to engage with people, she wouldn’t get angry with other residents because she can’t hear what they’re saying and she gets frustrated because she can’t understand what you’re saying properly. So, her quality-of-life will improve, even if it is for a short amount of time. – Care Assistant-1 (8 years in profession)	88.9%	Education & Persuasion	Providing information or case studies about the consequences of untreated hearing loss in residents with dementia to LTCH staff.
Physical Opportunity	Environmental Context and Resources	Poor Collaborations Between LTCHs And Audiology Services (Barrier)	They [audiology department] always want the resident to go to the hospital to have the hearing test. And that’s not always possible, especially if you’ve got someone that has got dementia who doesn’t do well with going outside in new environments, a noisy environment. – Manager (11 years in profession)	88.9%	Environmental Restructuring	Increasing number of staff on shift on days when residents have external healthcare appointments so that residents can be accompanied by LTCH staff. Ensuring audiology appointments are flexible and take place within the LTCH where possible.

### Knowledge

#### Lack of knowledge about hearing loss and hearing support (Barrier)

Most participants reported lacking knowledge of hearing loss and how best to manage residents’ hearing difficulties, placing emphasis on their variable knowledge of hearing aids. Only one senior carer and one LTCH manager believed themselves to have this knowledge but indicated strongly that their co-workers did not. LTCH staff often sought help from each other with hearing devices. Every participant revealed a lack of training on hearing loss and hearing care/support within their workplace. All but one expressed the desire for training and development to improve their awareness.

What I’ve learned from hearing aids is just picked up from other staff members or the nurses. We don’t really have any kind of formal training, or anything that I can think of where I could refer to… like any company policy kind of thing to say this is what we do with hearing loss. (Therapy Assistant, 1.5 years in profession)

We’re just winging it and hoping that what we’re doing is the best… not that any professional has told us that’s going to help that person but because we’ve had to try and find a way to communicate with someone. (Care Assistant-1, 8 years in profession)

Is it just my lack of knowledge or is it just that everybody doesn’t seem to know anything?(sic)… I’ve chatted to a few people in the care home… everyone seems to be the same as me. (Nurse-1, 23 years in profession)

### Beliefs about consequences

#### Recognition that untreated and under-treated hearing loss is detrimental to residents with dementia (Facilitator)

Ninety percent of participants (all but one care assistant) discussed the negative consequences of residents not receiving adequate hearing support. Examples centred around social withdrawal, disengaging from activities, loneliness, upset and challenging relationships between residents. Increased agitation and aggression in residents with untreated or under-treated hearing loss exacerbated the stress of LTCH staff. For participants, regardless of job or LTC, these adverse experiences were motivation to providing hearing support in future.

If somebody is frustrated and they’re not hearing, with dementia patients it can make them quite aggressive, you know? They get aggressive with you. Like it’s you that’s causing that pain in their head. (Care Assistant-3, 1.5 years in profession)

But until it’s [hearing] taken away from you, you don’t realise how much it has an impact on everything that you do… The joy of listening to music, people really take it for granted, but if you can’t listen to music, then the emotion has gone. (Manager, 11 years in profession)

#### Recognition that providing hearing support is beneficial to residents with dementia (Facilitator)

The benefit of hearing support was a motivational factor for staff. All discussed the positive impact on social wellbeing following effective hearing support. Staff placed importance on their view that providing valuable person-centred care is dependent on good communication with residents. Four participants, with multiple years in LTC, viewed the ability to communicate with residents and hearing loss as a vital, but often difficult, part of caring, essential to giving residents choice and involvement with their own care.

I think there’d be more choice… we would help the residents to feel heard, which for me, that’s just crucial… I very often see that people don’t get relief until they get heard. Whether that’s their emotions, their thoughts, their feelings… (Mental Health Nurse-1, 26 years in profession)

When you build that communication, then you can start caring. Otherwise, how can you start caring, if you cannot connect or communicate with the person? So, it is a high priority for me. (Senior Carer-1, 17 years in profession)

If somebody is hearing properly, then they’re understanding, and if somebody understands something, then there’s less fear. And if there’s less fear, there’s going to be less aggression. So, on that principle, yeah, I’d say it really does help if somebody has got the appropriate apparatus to hear. (Care Assistant-3, 1.5 years in profession)

### Environmental context and resources

#### Poor collaborations between LTCHs and audiology services (Barrier)

All participants stressed the need for improved collaborations with audiology services. In their opinion, audiologists rarely visit LTCHs compared to other healthcare professionals. Two care assistants reported never having seen an audiologist during their time in the caring profession, and five explained the rarity of seeing an audiologist in the LTCH.

I’ve not met an audiologist in the care home situation… I think it’s terrible actually. (Care Assistant-3, 1.5 years in profession)

Long wait times for appointments were reported by senior staff members (nurses, management). The lack of available audiology appointments meant that residents were without working hearing aids, hearing aid batteries, access to hearing tests and earwax removal. This disjointed working relationship meant that a continuous hearing support package was not always possible.

When they [resident] come here, we straight away call the GP to refer the audiologist and sometimes it’s quick and sometimes it takes time. Months even. Not weeks… Resource-wise, in my home and the home I worked… we will (sic) lack. (Senior Carer-1, 17 years in profession)

Resource wise, again, there’s (sic) struggles that we have with audiology is difficult because if you do have one [hearing aid] that’s broken, it’s getting it fixed, it’s how quickly you can get it fixed. And getting it to them and from them, that’s staff out of the building or it’s me running around normally in my car with a bag full of hearing aids. (Manager, 11 years in profession)

Difficulties co-ordinating and facilitating audiology appointments for residents were highlighted by four participants, mostly those in senior roles. Staff believed residents to have disadvantaged access to audiology because they live in LTC, not the community. For example, expectations for residents to attend audiology clinics outside of the LTCH causing stress and confusion for residents. Transportation for residents with a staff member was difficult, as accompanying a resident to an appointment means being away from the LTCH for several hours, potentially leaving other residents with fewer caregivers.

Leaving the home to have that test… it’s too much. It takes too much from them than it gives back. (Mental Health Nurse-1, 26 years in profession)

We’ll then ring… the audiology department to explain “the individual that we’re dealing with has severe dementia, is there any chance you can come and perform the hearing test here? because if we took them to a hospital, it’s a very scary environment and they might not understand what’s going on”… We can’t really send carers all the time because it then impacts the rest of the residents. (Care Assistant-1, 8 years in profession)

### Optimism

#### Despondency about audiology services for residents with dementia (Barrier)

Due to fragmented collaborations between LTCHs and audiology, most participants (60%, independent of role or workplace) felt pessimistic about arranging appointments for residents. There were often misunderstandings and tensions between LTCH staff and audiologists about the need for services to be dementia-friendly: Staff argued for LTCH-based appointments and flexibility in assessments and management of hearing loss. This lack of flexibility resulted in senior LTCH staff being less likely to organise appointments for residents in future.

She [audiologist] wasn’t prepared to listen to where this man was with his dementia and some of the difficulties associated with that… she didn’t really understand how dementia can also play a part in hearing loss. (Mental Health Nurse-1, 26 years in profession)

Audiology departments don’t realise how stressful it is working with people with dementia and hearing loss. It kind of makes everything ten times harder than it already is. (Care Assistant-1, 8 years in profession)

They [audiology] don’t always understand because you say then “they won’t wear it [hearing aid]” or “they don’t like it” and it’s like “oh what do you want me to do? I’ve done the mould” and that’s it. They’ve done their job and they just leave it. (Manager, 11 years in profession)

The working relationships with audiology often left LTCH staff frustrated.

It’s just that lack of support and feeling alone when having to deal with situations like this. We can put as much stuff into place as we can to make everything easier, but we’re not experts in this field… You kind of get to the point where you’re like “what is the point?” (Care Assistant-1, 8 years in profession)

It always seems to be quite a fight to get them to do a home visit instead of them [resident] going to the hospital. I don’t think they understand the logistics of trying to get a resident to the hospital. (Manager, 11 years in profession)

#### The practicalities of conventional hearing aids for residents with dementia (Barrier)

While all participants agreed that supporting hearing loss was beneficial for residents, there was apprehension about traditional hearing aids for this population. Difficulties related to residents misplacing, hiding, breaking or not adapting well to their hearing aids. Overall, the responses of many residents to hearing aids led to pessimism about their effectiveness and a lack of motivation to use them as a treatment for hearing loss within LTC in future.

Rather than have the like kerfuffle or trying to put it on him [resident] and the hassle… they’d rather just let him not have it because… it’s too much effort for them to put it on and for him to fight back, whereas they can just kind of not put it on and let him go about his day. (Therapy Assistant, 1.5 years in profession)

We’ve had residents eating their hearing aids. That was a bit of a worry. Finding the battery after that had been chewed you think “oh no” if they swallow a battery that could obviously be quite serious. (Registered Nurse-1, 23 years in profession)

Difficulties tended to be more of an issue for residents with advanced dementia, compared to those with milder cognitive impairment, leading to frontline staff preferring alternative methods.

Other methods are definitely better. Just the loss of the hearing aid, it can create havoc. Especially if you’ve got very tenacious relatives. (Care Assistant-3, 1.5 years in profession)

With hearing aids, like they can die, they can get lost, they’re not that reliable, whereas communication cards are quite… they’re just easy and they’re quite accessible. (Therapy Assistant, 1.5 years in profession)

### Social/professional role and identity

#### Feeling responsible for the provision of hearing support as a care worker (Facilitator)

All participants felt responsible for providing hearing support to residents because they believed it to be within their job remit as care worker and because they identified as a caring individual.

It’s part of residents’ care, isn’t it? And if you’re not doing it, we’re falling short, aren’t we? So yes, it is a nurse’s responsibility. (Nurse-1, 23 years in profession)

I feel a bit responsible for putting a bit of pressure on audiologists, saying “Hiya. I really need you to come and see this lady that’s delusional, she’s hallucinating, she’s going through all this stuff” so I feel that that’s me. (Manager, 11 years in profession)

You’ve looked after some person and they’ve lost their hearing aid, you’re responsible because it was on your shift… the responsibility is with you the carer. (Care Assistant-3, 1.5 years in profession)

#### Lack of personal accountability for hearing support (Barrier)

Despite feelings of responsibility, there was a lack of personal accountability amongst staff for providing hearing support, addressed specifically by one participant.

I think staff need to take more of an onus on the responsibility for the hearing aids and whose job role it is, rather than just letting the resident try and find their own hearing aids. (Therapy Assistant, 1.5 years in profession)

While seven participants of various roles explicitly reported hearing support to be “everybody’s job”, this was not always productive, as hearing can be easily overlooked.

It should be everyone’s responsibility. (Care Assistant-2, 16 years in profession)

One nurse believed that having designated Hearing Champions would be beneficial, as responsibility for this aspect of care was not specified in their workplace.

A champion in the care home that they trained up… and everybody in the care home knew this carer or this nurse is the person that knows about hearing aids, and any questions that they’ve got they can refer to them. (Nurse-1, 23 years in profession)

## Discussion

This study identifies targets for behaviour change interventions to improve the provision of hearing support for LTCH residents, through exploration of the barriers and facilitators. Multilevel barriers were identified, from gaps in personal knowledge to systemic difficulties within the LTC sector. This is the first UK-based qualitative study to conduct a holistic investigation of the difficulties in providing hearing support to residents using the BCW [21].

Five prominent Theoretical Domains were identified, emphasising the complexity of providing effective hearing support to a population with additional support needs in the unique context of LTC. These results provide an evidence-base for targeting the capabilities, opportunities and motivation of LTCH staff in future hearing-related interventions for residents with dementia and hearing loss.

### Barriers and Facilitators to provision of hearing support to residents by LTCH staff

#### Knowledge

Staff lacked awareness (*Psychological Capability*) of hearing support, primarily focusing on hearing aid management, which was more common for those in more junior positions. This finding is consistent with existing evidence of variable knowledge of hearing loss in LTCHs internationally [15,18,19]. A lack of training and learning opportunities (*Education, Training*) in this area was evident. Participants wanted formal training and learning opportunities on hearing support to be provided, rather than learning non-evidence-based techniques from colleagues. Training and education on hearing support may not only boost knowledge but also confidence and autonomy within the workplace [4]. Currently, there is no mandatory training on hearing loss for UK LTCHs. This is possibly because hearing is seen less of a care priority compared to other needs e.g., dysphagia or mobility [19,31], therefore is not a priority for training providers with limited funds. However, the provision of basic hearing support training, including hearing aids, communication techniques and other tools such as flashcards, may positively impact residents with hearing loss who rely on staff to have and use this knowledge. Our findings suggest that it would be beneficial to include hearing support in basic training packages for LTCH staff.

#### Beliefs about consequences

Despite this lack of knowledge, all staff spoke of the positive consequences of providing hearing support, and the negative consequences of unsupported hearing loss in residents (*Reflective Motivation*). These perceptions were particularly motivating for care assistants, who experience the stresses of responsive behaviours in their day-to-day role [32]. In contrast with reports that staff view hearing as a very low priority, therefore being overlooked [19], our results suggest that LTCH staff are motivated to provide hearing support.

Recent systematic research has found hearing support to be effective in improving several outcomes for LTCH residents [13], mirrored by reports in the present study. Furthermore, participants also spoke of how the ability to communicate well with residents facilitated more personal, empathetic care provision. Adequate hearing support may allow residents to better understand and engage in discussions about their care. These results add to the literature exploring the effects of hearing loss on person-centred care within LTCHs, the gold standard for ensuring care reflects residents’ needs and preferences [8,33].

#### Social/professional role and Identity

The themes identified under this Domain (*Reflective Motivation*) were contradictory: Feelings of responsibility for hearing support as a care worker cf. the lack of personal accountability. Many participants overtly stated that hearing and communication support is the responsibility of *all* staff because they work in the care profession. However, this was not necessarily beneficial, and experiences shared appeared to contradict the intention, e.g., hearing devices going unchecked, batteries not being replaced, and family taking responsibility instead. Although collaboration between LTCH staff and family caregivers can be beneficial to residents’ wellbeing [34], the reasons why family might take ownership of care must be considered e.g., lack of staff knowledge or resources.

“Hearing Champions” have been recommended for improving hearing support via ownership and leadership [35]. However, implementation of the “Hearing Champion” across LTCHs is unclear and the long-term impacts of the role are unknown [19]. Although the “Champion” role has been successfully embedded in interventions for people with dementia [19,36], it has been criticised for its unclear expectations and the lack of requisite formal qualifications [37]. *Incentivisation* (e.g., monetary), alongside *Modelling* for other LTCH staff, may effectively improve uptake and engagement alongside the usual workload, as per the Behaviour Change Wheel [21]. LTCHs do not typically provide incentivisation, thus potentially hindering the motivation of staff to engage with a “Hearing Champion” role.

#### Environmental context and resources

Poor collaborations between LTC and audiology services (*Physical Opportunity*) were strongly emphasised. Participants reported routine audiology appointments to be uncommon, comparable to a UK-based survey on hearing healthcare within LTCHs [14]. Appointments were seemingly made reactively rather than proactively. Reports regarding referrals by LTCHs to audiology have been conflicting: Bott et al. [16] found that staff did not to refer residents to audiology services, however, Leroi et al. [38] showed that LTCH managers do refer residents (as did managers in the current study). Discrepancies may be due to LTCH role responsibilities; managers are generally responsible for liaising with external services, while junior staff are not. The qualitative approach used in the present study allowed further investigation of this matter, highlighting how the issues extend further than referrals and include the suitability of standard audiology services for residents.

In the UK, the inequitable and poorly co-ordinated access to national healthcare services for LTCH residents remains an ongoing issue [39]. This is the first qualitative study to specifically focus on audiology, and the effects this has on residents’ wellbeing. Not only were appointments for residents difficult to obtain, but in most cases, they took place in a hospital or clinic. For many residents, this is either difficult or impossible due to mobility problems, anxieties and distress in unfamiliar environments. Furthermore, managers and senior staff found arranging transportation challenging, consistent with Pryce and Gooberman-Hill [12]. An additional barrier is the need for a caregiver to accompany residents to appointments. Residents without involved family are therefore disadvantaged, reliant on the LTCH to have resources to facilitate transportation. However, when staff leave the LTCH to do so, this can impact on other residents who require care, adding to prevalent staffing issues [40].

#### Optimism

Staff were generally pessimistic about audiology services (*Reflective Motivation)* as, in their opinion, the services are inaccessible and unaccommodating for residents. Participants disclosed situations with clear tensions regarding what they believed were audiologists’ underestimation of the difficulties that residents with advanced dementia experience.

Greater co-operation between LTCHs and audiology services is required *(Environmental Restructuring)*, so that residents have equitable access to healthcare services, ideally within the LTCH. The Enhanced Health in Care Homes framework [41], a new model included in the NHS Long Term Plan, aims to improve multidisciplinary healthcare provision across LTCHs in the UK, including holistic assessments on admission and weekly “home rounds” from requisite multidisciplinary teams. Whether this framework has begun to or will improve, access to audiological services for residents is unclear, and follow-up research in the coming years is essential in understanding its effectiveness.

Finally, participants were apprehensive (*Reflective Motivation*) about the use of traditional hearing aids to manage residents’ hearing loss. Staff tended to prefer alternative methods, as discussed in previous studies [8,16], such as communication techniques or flashcards. Participants questioned the effectiveness and usefulness of hearing aids for those with more advanced dementia. Although hearing aids may improve residents’ ability to hear, they may not improve their ability to comprehend what was said; a difficulty associated with dementia [42]. Pessimism in these cases was accompanied by valid examples and experiences. Further research is required to understand the suitability of hearing aids for people with advanced dementia. Insight into which types of hearing aid, and under which environmental condition, are beneficial for people with dementia may increase uptake and reduce the apprehension felt by caregivers. Flexibility, adaptations and choice of hearing support is necessary for residents [4,13] and multi-component interventions using amplification, either via hearing aids or other hearing devices (personal sound amplification products), and communication techniques would be best suited.

### Strengths and limitations

A strength of this study is the participant diversity across several demographic factors (job title, experience, LTCH size, type and registration). A purposive sampling method was used, and there is confidence in the representativeness of UK-based LTCH staff [43]. Furthermore, the consistency in responses, which guided the decision to end data collection at ten participants, provides evidence that the barriers and facilitators identified are not specific to one region of the UK, one type of LTCH or one job role. The relatively small number of participants in this study could be considered a limitation. However, here we used information power (richness and quality of data, consistency and relevance of responses) to guide the decision to end data collection (as *recommended* by Braun and Clarke [44]). Furthermore, similar interview studies with ten participants have been successful in making recommendations for clinical practice using the TDF [45,46].

### Summary

The provision of hearing support for residents is complex. This study identified five TDF domains, mapping to three COM-B domains, categorising the multi-level barriers and facilitators. This is the first study to use the BCW [21] to understand what needs to change and provides exemplar interventions to address key issues. Interventions aimed at improving the effectiveness and suitability of hearing support for residents should be multi-faceted, targeting the capabilities, opportunities and motivations of LTCH staff. Targets for interventions include Hands-on training for staff in managing hearing devices and information on the consequences of unsupported hearing loss, appointing incentivised Hearing Champions to take ownership of hearing support, providing dementia-friendly adaptations to hearing devices, and ensuring that audiology appointments take place in LTCHs, where possible.

## Data Availability

Although every effort has been given to fully anonymise participant data for this study, much of the qualitative transcripts are sensitive and certain aspects may still be identifiable.

## Appendix

**Table:** Appendix A. Interview schedule.

Question	(If relevant) COM-B and *Theoretical Domains Framework* prompts
1. Please can you introduce yourself and say how long you have been working in care homes?	
2. How is hearing loss support provided for residents with dementia in your care home?	
General views on hearing loss support:	
3. What do you think about the quality of hearing loss support in care homes for residents with dementia? • Do you think that it is done well? Why/why not? 4. How easy do you think it is to provide hearing loss support for residents with dementia? • What impact do you believe that it has?	Reflective motivation *Beliefs about consequences*
Responsibility to provide hearing loss support:	
5. Do you see hearing loss support as something you are personally responsible for? • If not, who is and why? 6. Is hearing loss support a priority for you as a Care Assistant/ Registered Nurse etc. (delete as appropriate)? 7. What are the benefits of providing hearing loss support? • To residents with dementia? • To you as a Care Assistant/ Registered Nurse etc.? 8. What do you think are the drawbacks, if any, for not providing hearing loss support to residents with dementia?	Reflective motivation *Social/professional role and identityOptimismSocial/professional role and identityBeliefs about consequences*
9. Would you say that you are in the habit of providing hearing loss support for residents with dementia? • If not, what would be helpful in developing a routine for this? 10. How is hearing loss support prioritised compared with other aspects of care, for example hydration and skin integrity? • Can you explain why/why not?	Automatic motivation*ReinforcementEmotionIntentions*
11. What provides you with the ability to provide hearing loss support for residents with dementia in the care home? • To give you the knowledge, educationand awareness?	Psychological capability*KnowledgeSkills*
Current knowledge and training needs	
12. To what extent do you have the physical capability to provide hearing loss support? • For example, the *skills* to change hearing aid batteries or use loop systems. 13. What training/learning opportunities are available for hearing loss support? • Would you like more? Would you change this?	Physical capability *Skills*
14. What are the main challenges to providing hearing loss support for residents with dementia? • Do you think that there are differences in this between residents with dementia compared to residents without dementia? • What do you think is the best way to provide hearing loss support for residents with dementia?	
15. Do you receive support from or work collaboratively with other staff members to provide hearing loss support for residents with dementia? • From external services such as General Practitioner or audiologists regarding hearing loss support? • How do these arrangements work?	Social opportunity
16. To what extent does your workplace provide you with opportunities to provide hearing loss support for residents with dementia? • Enough time, enough resources etc.	Physical opportunity *Resources*
Open questions:	
17. What, if anything, would make supporting hearing loss in residents with dementia easier for you as a Care Assistant/ Registered Nurse etc.?	
18. Is there anything you would like to add to this discussion?	
